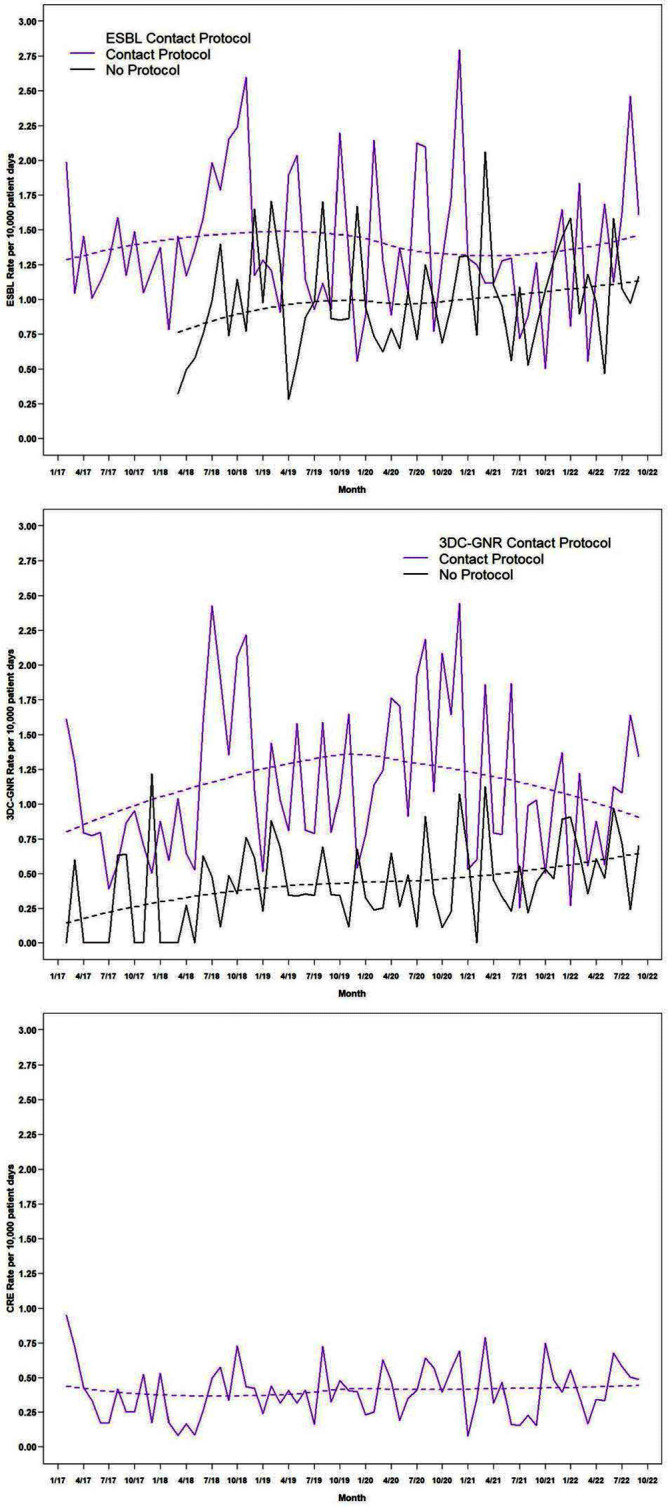# Impact of Discontinuing Contact Precautions for Multidrug-resistant Gram-negative Enterobacteriaceae in a Large Health System

**DOI:** 10.1017/ash.2024.310

**Published:** 2024-09-16

**Authors:** Sharon Karunakaran, Justin Ludwig, Christine Bridge, Elise Martin, Graham Snyder

**Affiliations:** UPMC Childrens Hospital of Pittsburgh; UPMC; VA Pittsburgh Healthcare System; University of Pittsburgh

## Abstract

**Background:** Contact precautions (CP) to prevent transmission of multidrug-resistant gram-negative (MDRGN) Enterobacteriaceae are recommended, although studies of discontinuation of CP (DcCP) have found no change in healthcare associated infections (HAI) due to extended-spectrum beta-lactamase (ESBL) producing Enterobacteriaceae. Limited data exists on DcCP for MDRGN in a large health system. **Methods:** We performed a retrospective observational study analyzing the relationship between use of CP and HAI due to two definitions of MDRGN Enterobacteriaceae: ESBL, and non-susceptibility to ≥3 drug classes (3DC-GNR), with carbapenem resistant Enterobacteriaceae (CRE) serving as control. The study included all inpatient admissions from 2/2017 through 9/2022 at 21 acute care hospitals. Hospitals had latitude to determine CP practices based on local risk assessment, but in 2/2018, system-wide transmission-based precautions guidance was updated to recommend DcCP for MDRGN Enterobacteriaceae and in 12/2019 was updated to clarify DcCP specifically for ESBL and 3DC-GNR while continuing CP for carbapenem-resistant organism carriage. We interviewed infection preventionists to define when CP were used for CRE, ESBL, and 3DC-GNR Enterobacteriaceae. HAI were defined using National Healthcare Safety Network criteria including all HAI categories. We compared the incidence rate of HAI attributable to the two MDRGN types in hospital months with and without use of CP, with HAI due to CRE as a comparison group since all hospitals used CP for CRE throughout the study period. **Results:** The periods of CP use, by hospital, are shown in Figure 1. Throughout the study period, there were 987 HAI attributed to ESBL Enterobacteriaceae, 579 due to 3DC-GNR Enterobacteriaceae, and 329 due to CRE. Figure 2 shows the unadjusted aggregate rate of HAI for each of the three MRGN types, including among hospitals with and without CP in each month, for ESBL and 3DC-GNR. In months with and without CP, the rate of HAI was 1.482/10,000 and 1.093/10,000 patient days (incidence rate ratio [IRR], 1.356 [95% confidence interval, 1.195-1.540]) for ESBL Enterobacteriaceae. In months with and without CP, the rate of HAI was 1.071/10,000 and 0.493/10,000 patient days (IRR,2.173[95% confidence interval, 1.838-2.569]) for 3DC-GNR Enterobacteriaceae. **Conclusion:** DcCP was not associated with an increase in HAI due to ESBL and 3DC-GNR Enterobacteriaceae in aggregated facilities that self-selected for DcCP. Facilities that used CP were associated with significantly higher rates of ESBL and 3DC-GNR Enterobacteriaceae, a relationship that did not change as hospitals DcCP for these MDRGN. Further analyses are necessary to assess for a causal relationship.

**Figure:**
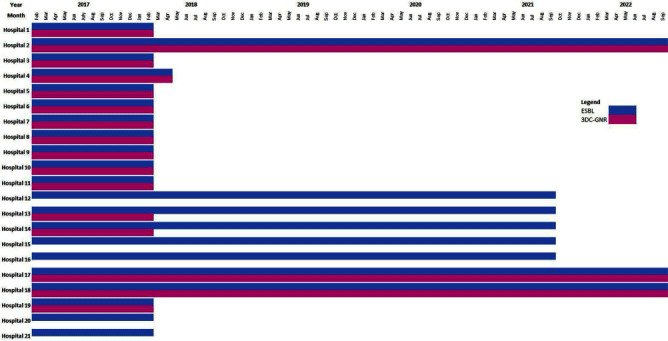

**Figure:**